# Life Review in Dementia: The Value of Identity

**DOI:** 10.1093/geroni/igaa057.879

**Published:** 2020-12-16

**Authors:** Diane Walsh, Hanna Ulatowska, Tricia Santos, Sara Aguilar, Rebecca Patterson

**Affiliations:** 1 The University of Texas at Dallas, Richardson, Texas, United States; 2 The University of Texas at Dallas, Dallas, Texas, United States

## Abstract

The stories told by veterans with dementia provide new insights into person-centered care for healthcare professionals. In this qualitative exploratory study, life review in seven WW II veterans with mild-moderate dementia was examined. Participants with mild-moderate dementia were selected from a larger study of seventy oldest-old veterans. Semi-structured interviews were conducted to elicit testimonial language about memorable war experiences, including their perception and evaluations of the war. The interviewer provided questions and prompts to facilitate responses when necessary. Despite decreased declarative memory manifested via reduction in details and specificity of information within narratives, participants demonstrated a desire to share their war time experiences. Veterans selected appropriate memories to share, indicating their preserved sense of self. They provided a general evaluation of their wartime experiences when prompted. Veterans expressed various types of identity related to both their social origin and their experience of participating in the war. Identities of social origin revealed cultural identities which were expressed via their sense of humor. Wartime identities include being a survivor, a patriot, and a tolerant person. Veterans also described how the GI Bill helped them become college educated. This study suggests that identity is relatively preserved more so in individuals with mild versus moderate dementia when producing autobiographical stories. The process of life review brings identity to the surface. Acknowledging the identity of individuals with dementia is essential to care as it recognizes the value of the person. Additionally, eliciting significant autobiographical memories serves as a valuable means of social engagement and connection.

